# Compliance With the US Food and Drug Administration’s Guidelines for Health Warning Labels and Engagement in Little Cigar and Cigarillo Content: Computer Vision Analysis of Instagram Posts

**DOI:** 10.2196/41969

**Published:** 2023-03-14

**Authors:** Jiaxi Wu, Juan Manuel Origgi, Lynsie R Ranker, Aruni Bhatnagar, Rose Marie Robertson, Ziming Xuan, Derry Wijaya, Traci Hong, Jessica L Fetterman

**Affiliations:** 1 College of Communication Boston University Boston, MA United States; 2 Department of Computer Science Boston University Boston, MA United States; 3 Department of Community Health Sciences School of Public Health Boston University Boston, MA United States; 4 Department of Medicine University of Louisville Louisville, KY United States; 5 American Heart Association Tobacco Regulation and Addiction Center Dallas, TX United States; 6 Department of Medicine School of Medicine, Vanderbilt University Nashville, TN United States; 7 Evans Department of Medicine and Whitaker Cardiovascular Institute Boston University Chobanian & Avedisian School of Medicine Boston, MA United States

**Keywords:** tobacco, cigar, little cigar, cigarillo, Instagram, social media, influencer promotion, tobacco advertising, health warning, machine learning, computer vision, warning label, health label, health promotion, advertising, advertise, smoking, smoker, algorithm, visualization

## Abstract

**Background:**

Health warnings in tobacco advertisements provide health information while also increasing the perceived risks of tobacco use. However, existing federal laws requiring warnings on advertisements for tobacco products do not specify whether the rules apply to social media promotions.

**Objective:**

This study aims to examine the current state of influencer promotions of little cigars and cigarillos (LCCs) on Instagram and the use of health warnings in influencer promotions.

**Methods:**

Instagram influencers were identified as those who were tagged by any of the 3 leading LCC brand Instagram pages between 2018 and 2021. Posts from identified influencers, which mentioned one of the three brands were considered LCC influencer promotions. A novel Warning Label Multi-Layer Image Identification computer vision algorithm was developed to measure the presence and properties of health warnings in a sample of 889 influencer posts. Negative binomial regressions were performed to examine the associations of health warning properties with post engagement (number of likes and comments).

**Results:**

The Warning Label Multi-Layer Image Identification algorithm was 99.3% accurate in detecting the presence of health warnings. Only 8.2% (n=73) of LCC influencer posts included a health warning. Influencer posts that contained health warnings received fewer likes (incidence rate ratio 0.59, *P*<.001, 95% CI 0.48-0.71) and fewer comments (incidence rate ratio 0.46, *P*<.001, 95% CI 0.31-0.67).

**Conclusions:**

Health warnings are rarely used by influencers tagged by LCC brands’ Instagram accounts. Very few influencer posts met the US Food and Drug Administration’s health warning requirement of size and placement for tobacco advertising. The presence of a health warning was associated with lower social media engagement. Our study provides support for the implementation of comparable health warning requirements to social media tobacco promotions. Using an innovative computer vision approach to detect health warning labels in influencer promotions on social media is a novel strategy for monitoring health warning compliance in social media tobacco promotions.

## Introduction

In 2021, overall 5.2% of middle and high school students in the United States reported ever using cigars, representing 1,400,000 youths who ever experimented with cigar products [1]. Cigars were also the most commonly used combustible tobacco products among US high school students in 2021 [1]. That cigars surpassed cigarettes in becoming the most popular combustible tobacco products among non-Hispanic Black middle and high school students is concerning [1]. Compared to non-Hispanic White youths, non-Hispanic Black youths had greater odds of initiating tobacco product use via cigars [2]. On the contrary, non-Hispanic White youths were more likely to initiate tobacco use through e-cigarettes [2]. Youths who initiated tobacco use via cigars were also more likely to become current tobacco product users of multiple products than youths who initiated tobacco use via e-cigarettes [2]. Importantly, longitudinal research suggests that the use of cigars may be a predictor of marijuana initiation among young college students [3]. Thus, cigar smoking among youths not only presents a critical public health issue but also raises concerns about health equity in tobacco prevention and control.

Tobacco advertising plays an important role in shaping tobacco-related knowledge, attitudes, and behaviors among youths [4]. A substantial body of evidence links advertising exposure with key factors that lead to youth tobacco use, such as curiosity about smoking, tobacco brand awareness, positive attitudes, and intentions to smoke [5-7]. In the United States, the tobacco industry has shifted its marketing efforts to the internet to circumvent restrictions on broadcast media (eg, TV and video), outdoor media (eg, billboards and public transit advertising), and tobacco product placement in entertainment media [8,9]. Specifically, in the United States, the 1998 Master Settlement Agreement prohibited tobacco companies from targeting youths in the advertising, promotion, or marketing of tobacco products [10]. The 2009 Family Smoking Prevention and Tobacco Control Act (Tobacco Control Act) established additional restrictions on youth-targeted tobacco marketing [11]. However, both the Master Settlement Agreement and Tobacco Control Act did not directly address social media–based tobacco marketing. As a result, the tobacco industry shifted its marketing efforts from traditional media forms such as print and billboards to the internet [12,13]. Social media–based advertising is largely an unregulated environment for tobacco companies to reach and engage current and potential customers [14,15].

Tobacco companies promote products on social media mainly through 2 means: brand-owned pages and influencer promotions. An analysis of 112 leading brands of tobacco products found that most brands had pages on at least 2 of the following social media platforms: Instagram, Facebook, Twitter, YouTube, Pinterest, and Tumblr [16]. In addition, tobacco companies promoted products through paid “influencers” who have large social media–based followings [17]. Social media promotions may be a more effective means for influencing tobacco perceptions and use than traditional advertising such as TV and print media that provide no interactive features, as social media gives the audience more options for engagement and interactions with tobacco content [18].

Influencers discussing tobacco products have the potential to affect followers’ attitudes and product use [19]. Followers of tobacco influencers are younger, have lower education, and are more likely to report past month tobacco use than those who do not follow tobacco influencers [20]. Youths are especially vulnerable to social media–based tobacco marketing in part due to their high level of internet and social media use [21], with youths aged 13-18 years spending over 8 hours on the internet every day [22]. Exposure and engagement with social media–based tobacco marketing, including social media promotions, are associated with tobacco product use among US youths [23,24].

Health warning statements serve as a source of health information, increase perceptions of risk, promote smoking cessation, and have the potential to lower smoking initiation among youths [25,26]. Health warnings are required to be displayed on the packaging and advertising for all tobacco products [27]. Even though the law also mandates the inclusion of health warning labels on cigars and pipe tobacco [28], the US District Court for the District of Columbia has issued an order vacating these requirements for cigar and pipe products. As a result, cigar and pipe tobacco firms may opt to voluntarily comply with the health warning provisions set by the US Food and Drug Administration (FDA). However, the 7 largest cigar companies in the United States must still display health warnings in both their advertising and packaging due to an existing consent agreement with the Federal Trade Commission [29]. These companies include Swisher International, Inc (producer of Swisher Sweets cigars) and Altadis U.S.A. (producer of Backwoods and Dutch Master cigars) [29]. The FDA mandates that health warning statements on advertising for covered, roll-your-own, and cigarette tobacco products must (1) appear on the upper portion of the advertisement within the trim area and (2) occupy at least 20% of the area of the advertisement [30]. However, social media advertising, including influencer promotional posts, has not been specified in the health warning requirements for any tobacco product.

Instagram is one of the most popular social media platforms among youth, with 72% of youths reporting Instagram use in 2018 [21]. The photo-oriented nature of Instagram makes it an ideal platform for influencer promotions, since images convey more emotions and intimate feelings than text-oriented platforms such as Twitter [31]. Health warning labels in celebrity-endorsed e-cigarette Instagram advertisements decreased viewers’ intention to use e-cigarettes [32]. Tweets that include health warnings for e-cigarettes were found to elicit more negative health perceptions of the e-cigarette brand than those without warnings [33]. The inclusion of FDA-mandated nicotine warning statements in Instagram e-cigarette promotions decreased the appeal of the posts [34]. However, little is known about the use of health warning labels in LCC influencer posts on Instagram and whether the presence of health warnings is associated with user engagement with LCC influencer promotions. This study identified LCC influencers using innovative methods and performed computer vision analysis to investigate the current state of LCC influencer promotions on Instagram and the use of health warning labels in LCC influencer promotional posts. We report here the development and use of an innovative computer vision method. We then use our computer vision algorithm to evaluate the effect of warning labels of leading LCC Instagram influencers on post engagement.

## Methods

### Data Collection

We focused on 3 LCC brands with the leading market shares in the United States, including Backwoods, Swisher Sweets, and Dutch Masters, which frequently feature influencers on their Instagram brand pages [35,36]. At the time of data collection, these 3 brands were also the most followed LCC brands on Instagram.

**Figure 1:**
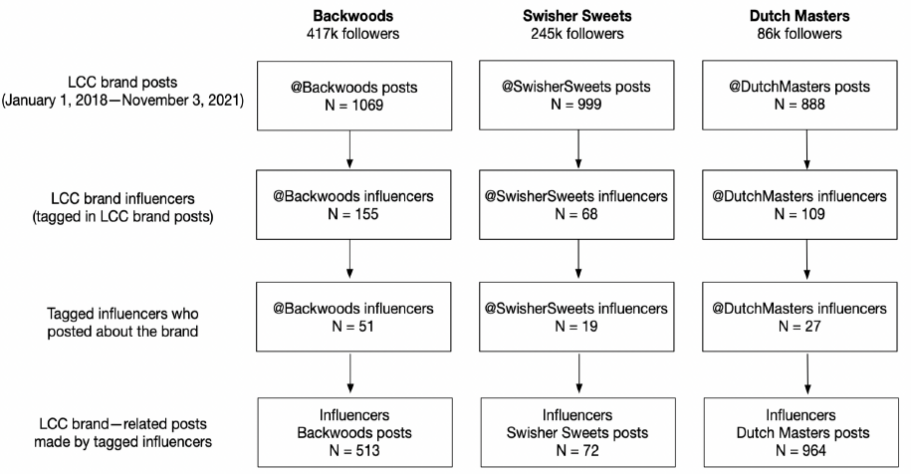
Data sampling procedure. LCC: little cigar and cigarillo.

In violation of Federal Trade Commission guidance [37], many influencers do not use methods to disclose that they have a “material connection” with the brand, such as including hashtags such as #ad or #sponsored. As a result, paid influencer posts can be difficult to identify and study. Thus, influencers were identified as individuals tagged by one of the 3 LCC brands. First, we scraped all Instagram posts from the 3 LCC brands, which were posted between January 1, 2018, and November 3, 2021. Then, we used the string-matching function in the R software to identify all handles tagged in the captions of the collected LCC brand posts. During this time, Backwoods tagged 155 unique Instagram users, Swisher Sweets tagged 68 Instagram users, and Dutch Masters tagged 109 Instagram users. We collected posts referencing LCCs published by each identified influencer from the users’ Instagram pages in November 2021. In total, we identified 51 Backwoods influencers who posted 513 Backwoods-related Instagram posts, 19 Swisher Sweet influencers who posted 72 Swisher Sweets-related Instagram posts, and 27 Dutch Masters influencers who posted 964 Dutch Masters–related Instagram posts (Figure 1). Pictures or videos of brand-related influencer posts were manually downloaded. Engagement metrics of each post, including the number of likes and comments, were also recorded. Collected data were stored in a password-protected computer and were only accessible to the authors.

### Manual Coding

Because not all influencer posts that mentioned brand names contained content related to cigar smoking or LCC brand–sponsored events, we manually coded whether the identified influencer LCC posts were relevant to cigar smoking and LCC brands. Two coders were trained to determine (1) if an influencer post was relevant (ie, it pertained to cigar smoking or LCC-sponsored events such as the Swisher Sweets Artist Project) and (2) among relevant posts, if the post contained a health warning. For video posts, coders watched the entire video and took screenshots if health warnings appeared.

To determine inter-coder reliability, 2 coders were trained on 20 images, and questions regarding coding criteria were discussed. Next, the 2 coders independently evaluated 150 posts (50 posts for each brand), after which Cohen kappa values were calculated to determine the intercoder reliability on the 2 coding questions. The average Cohen kappa values were 0.954 for question 1, that is, whether an influencer post was relevant (pertained to cigar smoking or LCC-sponsored events), and 0.966 for question 2, that is, whether the post contained a health warning (among LCC brand–relevant posts). The high Cohen kappa values indicate a high level of intercoder agreement between the 2 coders [38]. Two coders independently coded the remaining images in the sample.

### The Warning Label Multi-Layer Image Identification Algorithm

To determine the presence and properties of a warning label inside an image, we developed the Warning Label Multi-Layer Image Identification (WaLi) computer vision algorithm by integrating the computer vision library OpenCV [39] and the open-source OCR (Optical Character Recognition) engine Tesseract [40]. WaLi was developed to specifically identify compliance with 2 FDA guidelines for advertising of covered, roll-your-own, and cigarette tobacco products, which state that health warnings must (1) appear on the upper portion of the advertisement within the trim area and (2) occupy at least 20% of the area of the advertisement. In addition to detecting the word “warning” in the image, we also analyzed the image over 4 different levels: pixel color, pixel contours area, pixel contours shape, and text (using OCR). As a preliminary step, to preprocess the image and allow the algorithm to better detect the warning statement (if present), we applied a black and white color transformation and multiple blurring and morphological functions (erosion and dilation) to remove small noise components.

Since warning labels are required to have black borders, we applied a binary filter to select only the dark-colored pixels of interest and filter out all the others. The selected pixels were grouped into contours to analyze the shape and area. If the area of the contour was between 2 specified thresholds and the shape of the contour was described as a quadrilateral, the selected contour was passed to the last step of the analysis. The 2 area thresholds were selected using empirical experiments on the data available and on the basis of assumptions about how the warning label should look according to the FDA directives. In particular, in order to be able to track both valid and invalid warning labels (with respect to FDA regulations), we chose a minimum threshold of 600 pixels, which corresponds to the minimum size required for reading the words by the text OCR (more details about this method are provided in the next section) and a maximum threshold of half the image size.

Finally, we evaluated whether the image area identified by the contour contained the word “warning,” as mandated by the FDA for the health warning labels in advertising of roll-your-own and cigarette tobacco products [30]. We used the tesseract text OCR to extract the text content in the selected image area. If the keyword “warning” was found, then the algorithm returned the position of the warning label and area of the health warning as results. To increase the flexibility of this process, different variations of the described color filters and image preprocessing functions (including blurring and morphology) were used in the initial step of the analysis, allowing for the identification of nonstandard warning labels.

**Figure 2:**
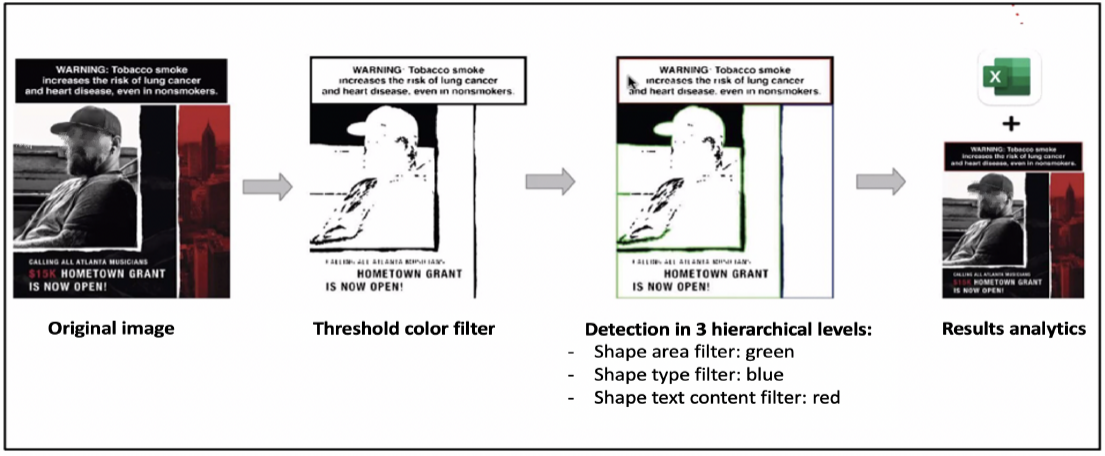
Computer vision process.

For all post images, the WaLi computer vision algorithm was used to determine (1) if the image contained a health warning, (2) the area (in pixel) of the warning label in relation to the image area, and (3) the placement of the health warning (upper portion of the image; Figure 2). In Figure 2, we highlight the 4 main steps of the warning labels’ detection process. The second image in the process describes the pixel color filter using binary thresholding with an example of the output given the original image as the input. The third image shows the output after combining the area, shape, and OCR filter (highlighted in the image with 3 different colors). Finally, the final output shows the original image with only the area that corresponds to the filter criteria highlighted in red. Our code is publicly available on GitHub [41].

### Statistical Analysis

We used negative binomial regression models to assess the association of health warnings with the engagement (numbers of likes and comments) of an LCC influencer post. Negative binomial models account for overdispersion in count data [42] and have been used in prior research evaluating social media engagement (eg, post “likes”) [43]. We also included influencers as random effects in the models as observations were nonindependent. Specifically, over half of the influencers published >1 LCC Instagram post. We also adjusted for follower counts and LCC brand, which can potentially affect the engagement of influencer posts on Instagram. Negative binomial models were fitted using the glmmTMB package in R (version 4.1.0; The R Foundation). The exponentiated regression coefficients in the negative binomial model are reported as incidence rate ratios (IRRs). Corresponding 95% CIs and 2-sided *P* values are also reported. Variance inflation factor scores for all independent variables were within the range of 0 to 2, indicating no substantive multicollinearity.

### Ethical Considerations

As only publicly available data were used, the author’s institutional review board determined that this study did not meet the definition of human participants research.

## Results

### Health Warnings in LCC Influencer Posts

**Figure 3:**
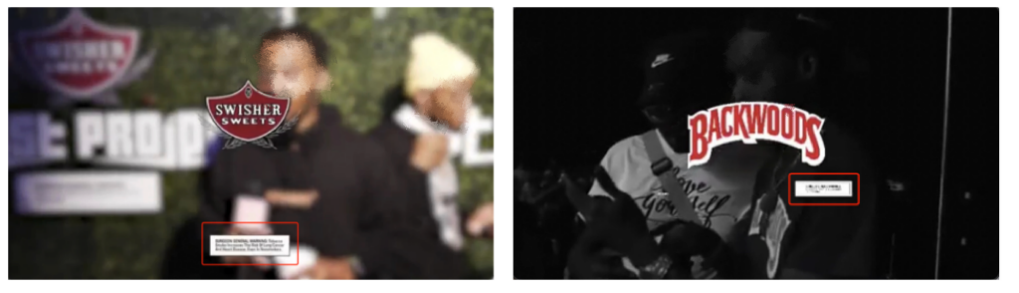
Examples of posts in which Computer Vision failed to detect the presence of health warnings.

In total, we identified 1549 LCC posts from influencers who were tagged by the 3 leading LCC brand Instagram pages. Manual coding revealed that 889 posts (470 Backwoods influencer posts, 60 Swisher Sweets influencer posts, and 359 Dutch Masters influencer posts) featured either cigar smoking or LCC brand–related events. Among the retained 889 LCC influencer posts, manual coding identified 79 posts that contained health warnings. When using the WaLi computer vision algorithm for the 889 LCC branded influencer posts, it successfully captured health warnings in 73 out of the 79 coder-identified posts. The computer vision method failed to detect health warnings in only 6 posts because the health warnings were either too blurry or too small (Figure 3). Thus, the overall accuracy of using computer vision to detect the presence or absence of health warnings in our data set was 99.3%. Subsequent analyses were based on the results reported by the computer vision analysis.

Only 8.2% (n=73) of the LCC influencer posts contained a health warning. Among these 73 posts, the health warning label occupied an average of 8.2% of the area of the post. Specifically, only 4.1% (n=3) of LCC influencer posts included a health warning that occupied at least 20% of the total post, as required by the FDA for advertisements of roll-your-own tobacco and cigarettes [30]. Only 23.3% (n=17) of the posts placed a health warning in the upper area of the post per FDA requirement. Overall, among the 889 identified influencer posts of LCCs on Instagram, only 1 (0.1%) fully met the FDA requirements for health warning labels in tobacco advertising for roll-your-own tobacco and cigarettes, containing a health warning that constitutes at least 20% of the area of the post, and placing the label in the upper portion of the post.

### Association of Health Warnings With LCC Influencer Post Engagement

In negative binomial analyses, influencer posts that contained health warnings received fewer likes (IRR 0.59, *P*<.001, 95% CI 0.48-0.71) and fewer comments (IRR 0.46, *P*<.001, 95% CI 0.31-0.67) than those that did not contain health warnings, after adjusting for the number of account followers and LCC brand. Holding the number of followers and LCC brand constant, the presence of a health warning in an LCC influencer post was associated with a 41% decrease in the rate of likes and a 54% decrease in the rate of comments.

## Discussion

Developing and using an innovative computer vision method—WaLi—our study evaluated the use of health warnings in LCC influencer promotions on Instagram and the association of health warnings with post engagement. We found that few LCC influencers’ promotional posts contained a health warning. Additionally, we evaluated the location and size of warnings in LCC influencer posts in accordance with the FDA's warning requirements for roll-your-own tobacco and cigarette advertising. Even though LCC influencer promotions currently fall outside the scope of the FDA health warning requirements, our findings reveal that a very small number of LCC influencer posts met the requirement for using health warnings that constitute 20% of the advertisement's area and are located in the upper portion of the advertisement. Notably, the presence of health warnings in LCC influencer promotional posts was associated with less post engagement, including fewer likes and comment counts.

The current federal laws require warnings on advertisements for tobacco products but do not specify whether the rules apply to social media advertising [29,30]. Our findings support the use of health warnings in tobacco branded content and influencer promotions on social media. We found that the presence of health warnings in LCC influencer posts was associated with lower post engagement (ie, likes and comments). Our research builds upon previous research, which demonstrated that exposure to and engagement with social media–based tobacco marketing, including social media marketing, is associated with tobacco product use among US youths [23,24].

Our study is among the first to demonstrate the relation of health warning statements with engagement of LCC-related content posted by influencers tagged by LCC brands. The LCC brands deliberately tagged an influencer account that has a wide reach on social media, which allows its users to engage with the tagged influencer account. It is possible for influencers to transition to brand ambassadors and establish a lasting partnership with a brand [44]. One example of this is Swisher Sweets and their use of the Artist Project to cultivate partnerships with various influencers within the hip-hop music industry [45]. Those influencers then generate complimentary branded content on their own Instagram pages and engage their followers with branded promotional content. Yet, very few LCC branded influencer posts contained a health warning. When health warnings were present, the posts were associated with less engagement. Given that compared to nonfollowers, followers of tobacco influencers are more likely to be younger and have a lower level of education, making the followers more susceptible to tobacco influencer promotions [20]. Cigar brands frequently employ influencers to market their products on Instagram, with the majority of the influencers being people of color from the music industry, who are particularly appealing to younger, African Americans [35]. Future research is needed to examine whether Black individuals are more likely to encounter LCC influencer promotions on social media, as well as the effects of such exposure on their beliefs and cigar use.

Our study suggests that the incorporation of health warning requirements in social media posts promoting tobacco products could help reduce engagement with promotions of tobacco products on social media. Future studies are needed to identify the most effective implementation strategy of health warnings in social media promotional posts of tobacco to decrease the use and uptake of tobacco products among youths [33]. For example, irrespective of whether the effect of health warning labels on tobacco perceptions and initiation depends upon the size and design [25], it is unclear if previous guidelines on size and design, which focused on health warnings for product packaging [46], are effective in a social media environment.

Our study has several limitations. We only analyzed 3 LCC brands on one social media platform (Instagram). Our findings may not be generalizable to other tobacco products and social media platforms. We also do not know the tobacco use status of the individuals who engaged with the LCC influencer posts; thus, we cannot demonstrate the causal effects of health warnings on the audience’s attitudes and behaviors toward LCCs. Future research is needed to investigate the effects of health warnings in social media promotions of LCCs on youths’ attitudes toward LCCs, their onset, and use. Lastly, despite the fact that the identified influencers were tagged by LCC brand posts, we cannot verify if those individuals are paid by the LCC brands. Influencers can be compensated not only monetarily but also through performance and collaboration opportunities. It is important to conduct further research on how the tobacco industry uses these strategies to attract influencers and promote tobacco products in order to inform future advertising policies in the digital age.

Our study used an innovative strategy to identify influencers who were tagged by the leading LCC brands. This method of identifying influencers can be used to study influencer promotions of other tobacco products such as e-cigarettes, which are frequently advertised on social media [47]. We also implemented a state-of-the-art computer vision method to detect and evaluate health warning statement properties in Instagram influencer promotions. Compared to traditional manual coding of images, WaLi is much more efficient at providing an accurate measurement of the size and location of health warnings in visual tobacco advertisements. Such efficiency and accuracy can be particularly useful for monitoring compliance with health warning regulations in tobacco advertising and for evaluating the effectiveness of such policies in reducing the appeal of tobacco products. Thus, WaLi can be readily used for policy evaluation and surveillance of health warning compliance in various social media–based tobacco advertisements such as email and website advertisements.

In conclusion, our study lends support for the requirement of health warning statements in brand-related influencer promotions of LCCs on social media. Extending health warning requirements to social media advertisements of tobacco products warrants further research.

## Abbreviations

FDA: US Food and Drug Administration
IRR: incidence rate ratio
LCC: little cigar and cigarillo
OCR: Optical Character Recognition
WaLi: Warning Label Multi-Layer Image Identification
